# Perception of overseas experiences among medical students in Japan: a national online survey

**DOI:** 10.1186/s12909-023-04384-0

**Published:** 2023-06-21

**Authors:** Junna Iwata, Ryota Todoroki, Takehiro Hashimoto, Misa Hyakutake, Harumi Gomi, Akira Nishizono

**Affiliations:** 1 Keio University School of Medicine, Tokyo, Japan; 2 Faculty of Medicine, School of Medicine, Oita University, Oita, Japan; 3 Department of Microbiology, Oita University Faculty of Medicine, Oita, Japan; 4 Infection Control Center, Oita University Hospital, Oita, Japan; 5 Medical Education Center, Keio University School of Medicine, Tokyo, Japan; 6 International University of Health and Welfare School of Medicine, Narita, Japan; 7 Research Center for Global and Local Infectious Diseases, Oita University, Oita, Japan

**Keywords:** Japan, Medical student, Work abroad, Study abroad, Medical education, Non-English-speaking countries, National online survey

## Abstract

**Background:**

Data on the perceptions of medical students on international experience in non-English-speaking high-income countries (HICs) are very limited. This study aimed to assess the perceptions of medical students in Japan toward overseas experience while in school and post-graduation, as well as to characterize the support they require to pursue their profession in international settings.

**Methods:**

A cross-sectional national survey was administered online between September 16, 2020, and October 8, 2020. Participants were recruited from 69 medical schools using snowball sampling through acquaintances and social media platforms. The survey results were analyzed by two researchers.

**Results:**

A total of 548 students from 59 medical schools responded to the survey. Among them, 381 respondents (69%) expressed interest in working abroad, while only 40% seriously considered it. The majority of students responded that they would like to pursue clinical training abroad for a short term or while they were medical students (54%) or during a residency/fellowship (53%). The most popular regions among the respondents for future international experiences were North America and Europe. Finally, the most reported reasons for hesitation to work abroad were language barriers (70%), followed by lack of clarity regarding career options after working abroad (67%), difficulties obtaining medical licensure abroad (62%), and the lack of role models (42%).

**Conclusions:**

Although nearly 70% of participants reported a high interest in working overseas, various barriers to working abroad were identified. Our findings identified key problem areas that could be targeted when promoting international experiences for medical students in Japan.

**Supplementary Information:**

The online version contains supplementary material available at 10.1186/s12909-023-04384-0.

## Background

Globalization has become increasingly important in the field of medical education. In the United States of America (USA), the Association of American Medical Colleges designates 15 core competencies for medical students. This includes “Cultural Competence,” which requires students to interact effectively with people from diverse backgrounds [1]. In Japan, the number of foreign residents has been increasing annually, and in 2022, it has reached a record high of nearly 3 million [2]. As the number of foreign residents increases, there is an increasing need for global human resources that can handle a variety of patients. The Model Core Curriculum for Medical Education in Japan notes “playing social roles required as a health professional and contributing to local and international communities” as one of the “Basic Qualities and Abilities Required of a Physician” [3]. It is evident that global medical personnel with a broad perspective are needed around the world.

Globalization has provided medical students with increasing opportunities to communicate across borders both in-person and online, while in school. These experiences include internships, study visits, project work, language courses, and summer schools [4]. International health electives (IHEs) in medical schools are good examples of effective global health education. IHEs provide students with extensive opportunities to reflect on their personal and professional identities [5] and can influence their final career decisions, all of which can have a life-changing impact on future physicians [6].

Some studies have examined the preference for overseas experiences among medical students from English-speaking high-income countries (HICs) and their attitudes toward IHEs. Students from low- or middle-income countries (LMICs) often pursue overseas careers in HICs because of better work conditions with higher income, larger capacity, and advanced training opportunities in those countries [7–9].

However, few studies have examined the attitudes of medical students from non-English-speaking HICs toward overseas experiences other than studying abroad and IHEs while in school, not only while in school but also after graduation [4, 10, 11].

Japan is one of the largest non-English-speaking HICs. Most students in Japan enroll in medical schools immediately after graduating from high school. With the exception of certain cases such as transfer programs, medical school education is completed within six years. This style has been adopted in Germany, the Netherlands, Spain, Korea, and Singapore [12]. In general, the first two years are designated for liberal arts education, the next two for preclinical studies, and the final two for clinical clerkships. As of 2022, Japan has 82 medical schools (51 public and 31 private schools). Each year, these schools enroll approximately 9,000 students across the country [13]. There are no consistent national guidelines specifying the number of English classes or IHEs in medical-school curricula.

The Association of Japan Medical Colleges (AJMC) revealed that 58% of Japanese medical students would like to study abroad while in school or after graduation through a survey conducted in 2020 [14]. However, the number of students gaining experience abroad while in school was significantly lower than that in Europe and the US. More specifically, in Japan, only around 1% (around 750 out of 7,500 students) studied abroad between 2011 and 2013 [15].

Additionally, only approximately 20% of medical students in Japan reported being satisfied with the current study-abroad support system [14]. Previous studies have reported a lack of foreign language skills, concerns about life in foreign countries, and lack of friends as factors that discourage dental students in Japan from studying abroad [16]. Another concern for studying abroad is the cost burden associated with it [17].

As few large-scale surveys have been conducted to reveal the barriers to overseas experience among medical students in Japan, this study aims to assess the perceptions among Japanese medical students toward overseas experiences and characterize the support they require. We also aim to understand the barriers to converting interest into action for overseas experience among these students, which can be applied to other countries, particularly non-English-speaking HICs.

## Methods

### Study design

This cross-sectional study utilized online-based surveys with Google Forms™ as part of the projects of the Student Committee, American College of Physicians Japan Chapter. We developed an questionnaire to investigate the perceptions of Japanese medical students on overseas experiences during school and after graduation.

### Measures and operationalization

The questionnaire was titled “Survey of medical student attitudes toward overseas experience” and was developed in light of the stated objectives and purposes of the study. The experimental protocol was approved by the Ethics Committee of Oita University Hospital (approval number #2315) and the Keio University School of Medicine Ethics Committee (approval number #20221135). The questionnaire was self-administered, and no relevant information was collected. The participants were provided with the opportunity to opt out at any time.

The survey was designed by two authors and included 13 questions. Additional File 1 is the questionnaire used in this study. The survey inquired about the following: a) background: name of institution, school year, b) interest and intent level: 1 (not at all) to 5 (very high) using the Likert scale, c) barriers to working abroad, d) preparation status to work abroad, and e) desired career: types such as clinical training abroad, research abroad, working in international NGOs, clinical specialty (internal medicine, surgery, pediatrics, and others), and region.

### Survey distribution

The questionnaire was distributed amongst medical students in Japan between September 16, 2020, and October 8, 2020. The survey was distributed primarily in two ways. First, we used snowball sampling to reach out to a significant number of students who were interested in international activities. Second, we made use of a group in Slack®, a chat and messaging app platform, that included students across all 82 medical schools in Japan, to obtain questionnaire responses regardless of students’ interests. To prevent a single student from entering the survey more than once, the students had to register their email addresses when filling out the form.

### Statistical data analysis

All data were analyzed in RStudio (version 1.2.1335) [18] using basic R functions. Participants were divided into two groups based on their responses to questions about interest and intent level. The interest and intent levels for working abroad were measured using the Likert scale from 1 to 5, and a score above 4 was defined as a high interest or intent group. The pre-clinical group was defined as students in school years 1 to 4 and the clinical group as students in school years 5 and 6.

Correlations between the groups of students and their answers to specific questions were analyzed using Pearson's chi-square test and Fisher's exact test, where a *P* value < 0.05 was considered statistically significant.

## Results

### Characteristics of participants

A total of 548 students from 59 medical schools in Japan participated in this study. The distribution of their school years is presented in Table 1. The number of pre-clinical-year students (school years 1 to 4) was 413 (75%) and that of clinical-year students (school years 5 and 6) was 135 (25%).

**Table 1:** Distribution of school years of participants

School Year	Number (%)
1	127 (23)
2	82 (15)
3	106 (19)
4	98 (17)
5	87 (15)
6	48 (8.8)
Total	548

### Interest and intent level in working abroad

**Fig. 1:**
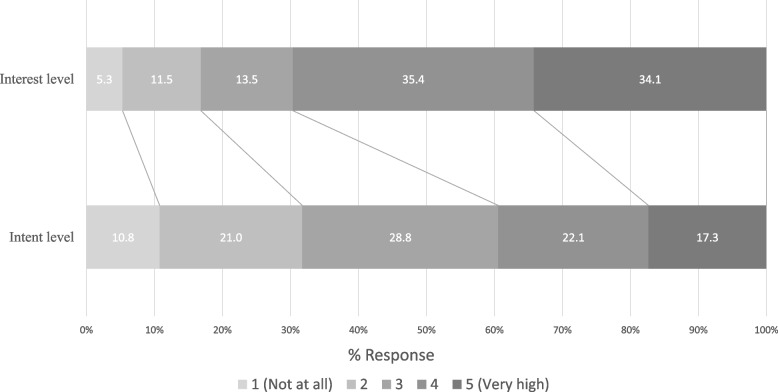
Interest and intent level in working abroad (*n* = 548)

Figure 1 shows the number of students who were interested in and seriously considered, working abroad. Nearly 70% of the participants were interested in working abroad with an interest level of 4 or higher. However, less than 40% seriously considered it with an intent level of 4 or higher.

Results of Likert-type questions (1: Not at all – 5: Very high) on interest and intent to work abroad. Participants with a score above 4 were defined as the high-interest or high-intent group.

### Barriers to working abroad

The most reported reasons for hesitating to work abroad included language barriers (70%), followed by lack of clarity regarding career options after working abroad (67%), and difficulties obtaining foreign medical licensure (62%).

The sub-analysis results for the high-interest group are presented in Table 2. Correlations between groups of 1) high interest and low intent and 2) high interest and high intent were analyzed using Pearson's chi-square test, where a *P* value < 0.05 was considered statistically significant. Language barriers remained the number one reason for all groups but were especially problematic in the high-interest and low-intent groups. The lack of role models was not elicited as one of the top three barriers in all participants but was reported to be so only among the high-interest group.

**Table 2:** Analysis of the responses to the question “What is your reason for hesitating to work abroad?”

	**Total**	**High interest**	**High interest and low intent**	**High interest and high intent**	***P*** ** value**
	**(** ***n*** ** = 548)**	**(** ***n*** ** = 381)**	**(** ***n*** ** = 165)**	**(** ***n*** ** = 216)**	
	**Number (%)**	**Number (%)**	**Number (%)**	**Number (%)**	
**Language barriers**	381 (70)	246 (65)	121 (73)	125 (58)	**0.003**
**Do not know other career options**	368 (67)	190 (50)	86 (52)	104 (48)	0.506
**Obtaining foreign MD** ^**a**^ ** licensure**	337 (62)	252 (66)	106 (64)	146 (68)	0.565
**Lack of role models**	230 (42)	190 (50)	86 (52)	104 (48)	0.506
**Cannot work in both domestic and international settings**	172 (31)	129 (34)	54 (33)	75 (35)	0.765
**Lack of peers who share the same goal**	136 (25)	114 (30)	41 (25)	73 (34)	0.076

High scores of 4 and 5, low scores of 1–3

^a^ *MD* Medical doctor

### Preparation status to work abroad

Table 3 shows how students in the high-interest group prepare to work abroad. Correlations between groups of 1) high interest and low intent and 2) high interest and high intent were analyzed using Pearson’s chi-square test, where a *P* value < 0.05 was considered statistically significant. Overall, the group with high intent did significantly more preparations than the group with low intent. “General English language study (any form of learning the English language except for professional English including medical English)” was the most common preparation. However, only 27% of the high-interest group studied medical English. Although 126 (33%) students gathered information from the Internet and other sources, only 63 (17%) gathered information by participating in lectures delivered by doctors with experience abroad.

**Table 3:** Sub-analysis among the high-interest group of the responses to the question “How are you preparing to work abroad?”

	**Total**	**Low intent**	**High intent**	***P*** ** value**
	**(** ***n*** ** = 381)**	**(** ***n*** ** = 165)**	**(** ***n*** ** = 216)**	
	**Number (%)**	**Number (%)**	**Number (%)**	
General English language study*	235 (62)	78 (47)	157 (73)	**< 0.001**
**Considering studying or training abroad**	132 (35)	35 (21)	97 (45)	**< 0.001**
**Collecting information from the Internet and other resources**	126 (33)	28 (17)	98 (45)	**< 0.001**
**Studying Medical English, for example, by preparing for USMLE®** ^**a**^	103 (27)	22 (13)	81 (38)	**< 0.001**
**Nothing in particular**	73 (19)	60 (36)	13 (6.0)	**< 0.001**
**Participating in lectures delivered by doctors with experience abroad**	63 (17)	11 (6.7)	52 (24)	**< 0.001**

High scores of 4 and 5, low scores of 1 to 3

^* ^General English language study includes any form of learning the English language except for professional English including Medical English

^a^USMLE® = the United States Medical Licensing Examination®

### Career of interest

The results for the types of careers in which the students were interested are shown in Table 4, divided into two groups: high and low interest. In total, more than half of the students responded that they would like to pursue clinical training abroad in the short term, while they were medical students or during a residency or fellowship. Even in the low-interest group, 40% answered that they were interested in clinical training abroad in the short term. Also, only 51 (9.3%) responded that they thought about working only in Japan, while the remaining 91% were considering working abroad in some way.

**Table 4:** Analysis of the responses to the question “Which type of international career are you interested in?”

	**Total**	**Low interest**	**High interest**	***P*** ** value**
	**(** ***n*** ** = 548)**	**(** ***n*** ** = 166)**	**(** ***n*** ** = 381)**	
	**Number (%)**	**Number (%)**	**Number (%)**	
**Clinical training abroad (short-term or during medical school)**	297 (54)	67 (40)	230 (60)	**< 0.001**
**Clinical training abroad (residency or fellowship)**	291 (53)	42 (25)	249 (65)	**< 0.001**
**International NGO **^**a**^**e.g. MSF **^**b**^ **, Red Cross**	189 (35)	31 (19)	158 (42)	**< 0.001**
**Conducting research abroad**	183 (33)	37 (22)	146 (38)	**< 0.001**
**International Organization** **e.g. WHO** ^**c**^ **, UNICEF** ^**d**^	156 (29)	16 (9.6)	140 (37)	**< 0.001**
**Health officer at the Japanese governmental organization** **e.g. Japanese embassies in foreign countries**	78 (14)	13 (7.8)	65 (17)	**0.007**
**Professional at the Japan International Cooperation Agency**	70 (13)	6 (3.6)	64 (17)	**< 0.001**
**Only thinking about working in Japan**	51 (9.3)	50 (30)	1 (0.3)	**< 0.001**

High scores of 4 and 5, low scores of 1–3

^a^ *NGO* Non-governmental organizations

^b^ *MSF* Médecins Sans Frontières

^c^ *WHO* World health organization

^d^ *UNICEF* United Nations international children’s emergency fund

Regarding the destination for an experience abroad, the majority of students showed a preference for developed countries such as North America (71%) and Europe (62%), while some students wanted to have their experience in Asia (32%), Oceania (23%), Africa (20%) and South America (10%). There were no statistically significant differences between the preclinical and clinical groups in terms of interest level, intent level, and types of careers they were interested in. The two most popular specialties chosen by students were internal medicine (70%) followed by surgery (62%). There were no statistically significant differences in interest level, intent level, or types of careers they were interested in among the four groups of desired specialties.

## Discussion

### Perceptions among the medical students

In our study, 70% of the participants had a high level of interest in studying or working abroad. This number is slightly higher than the nationwide survey in 2020, which showed that 60% of medical students in Japan were interested in studying abroad [14]. This difference can be attributed to selection bias as students who voluntarily answered this survey most likely had a relatively high interest in the overseas experience.

### Barriers to and preparations for working abroad

We found a striking gap between interest and intent among the students. Many students were interested in the overseas experience, but only some seriously considered it. The most reported reason for hesitation to work abroad was the “language barrier” (Table 2). This barrier is common among Japanese undergraduates and has also been reported in dental students [16, 19, 20]. Only 54% of medical students in Japan were satisfied with the number of hours of English language classes [14]. Most medical schools in Japan provide almost all classes and clinical clerkships in Japanese [20], and the number of hours of English language education depends on each medical school. To encourage more students to apply for international experiences, English language skill support will undeniably be effective. This may include more exposure to English through classes and seminars held in English and increasing the number of international students through student exchange programs [20].

This study also revealed that only some students studied medical English in preparation, whereas many medical students studied general English. There are some movements to promote medical English education in Japan [21]; however, the fact that only some medical students are eager to learn medical English is a challenge. Offering more learning opportunities focusing on medical English may be one of the solutions to the "language barrier," which is the biggest barrier to overseas experience for medical students in Japan.

Another suggested barrier was the lack of role models. According to a study targeting healthcare students, factors that support decisions to study overseas include having sufficient information about study abroad programs, especially early in an academic program, having an interest in other cultures/countries, and having academic staff and family as positive role models who motivate them to study abroad [17]. The lack of role models may be related to uncertainty about career options and lead to hesitation in going abroad. Several institutions and universities have held regular career seminars with doctors studying abroad [22]. However, the opportunities to discover role models for a wide range of career options are limited. Medical schools should provide more opportunities for students to seek mentorship, such as by inviting a physician working for an international Non-Governmental Organization to speak, which was the second most popular career after clinical training abroad in this study.

This survey was conducted in 2020, during the COVID-19 pandemic. It is interesting to note that even among the high-interest group, approximately 20% of students indicated that they were not particularly getting prepared. Our study showed that approximately 16% of medical students in Japan reported they did not study English in 2020, which was the highest number between 2016 and 2020 [23]. This may reflect the loss of motivation in students due to mobility restrictions caused by COVID-19. The results of the present study may, therefore, be related to the reduced motivation caused by the COVID-19 pandemic.

### Career of interest

Our study revealed that there is a high demand among medical students for studying abroad for a short-term while in medical school. According to the results of a 2011–2013 nationwide survey, 70 out of 80 universities across the nation sent only 700 to 800 exchange students abroad (< 1% of all medical students), and more than half of them went abroad for periods of two to four weeks [15]. Such short-term clinical training abroad is a highly valuable experience when students become doctors, as it promotes reflective self-relativization and has contributed to medical professional identity formation for more than a decade [24]. Another study suggested that greater exposure to colloquial English was one of the determinants of greater willingness among Japanese doctors to see foreign patients [25]. Although the number of medical students sent overseas has increased [26], short-term study-abroad programs for medical students should be further promoted.

The majority of participants wanted to have experience overseas in North America and Europe. This result is similar to the previous research, which found that approximately 70% of Japanese medical students were choosing to study in Europe and North America from 2011 to 2013 [15], and more than 60% of Japanese dental students expressed a preference for English-speaking countries, such as the US, the UK, and Australia [16]. The preference of Japanese students for these medically advanced countries may be due to their desire to learn medical skills while being exposed to the English language. Our findings revealed that some students were interested in studying or working in regions outside of North America and Europe. The leading destinations of medical students in Japan for international electives were Asia (36.4%), the United States (30.1%), and Europe (23.6%) in 2018 [26]. Japan needs to provide opportunities for medical students and doctors to gain experience and broaden their perspectives in various regions all over the world either during or after their studies, as the other HICs do [27]. Universities or institutions in Japan should actively promote collaboration not only in North America and Europe but also with other regions worldwide.

### Limitations

This study had some limitations. First, there were several types of selection bias, such as sampling bias and volunteer bias, because of the sampling method. This study originally started as a project of the Student Committee, American College of Physicians Japan Chapter, and the project itself was a pilot in nature. Thus, some information was not collected in this study, such as self-identified gender, age group, and financial situation, which could influence the career plans of the students. The financial situation affects students’ motivation to go abroad. A previous report targeting dental students in Japan indicated that household income was significantly correlated with concerns about overall expenses for studying abroad [16]. Although several foundations and universities offer scholarships for physicians and medical students to study or work abroad [15, 28, 29], the cost burden among them has not yet been revealed. It would also be helpful to conduct a qualitative analysis to obtain more information on the perceptions of the students.

## Conclusions

We conducted an online-based cross-sectional study to reveal the perceptions of and barriers to overseas experience among medical students in Japan. Participants had a high level of interest in working abroad (69% of participants). Short-term clinical training abroad was the most popular option for overseas experience among the participants and should be further promoted. Language barriers and a lack of role models contribute to the reluctance of students to work abroad. It is important to reduce these barriers for non-English-speaking HICs including Japan. Further studies should include more comprehensive items to identify the detailed needs and resolutions among the students.

## Supplementary Information

**Additional file 1.** **Questionnaire.** Survey of medical students’ attitudes toward overseas experience.

## Data Availability

The datasets used and/or analyzed during the current study are available from the corresponding author upon reasonable request.

## Abbreviations

HICs: High-income countries
LMICs: Low or middle-income countries
AJMC: The association of Japan medical colleges
IHEs: International health electives
US: United States
MD: Medical doctor
USMLE®: United States medical licensing examination®
WHO: World health organization
UNICEF: The United Nations international children's emergency fund
MSF: Médecins Sans Frontières
NGO: Non-governmental organization
