# Supplementary material for: Perception of overseas experiences among medical students in Japan: a national online survey
Source: BMC Med Educ. 2023 Jun 21;23:461. doi: 10.1186/s12909-023-04384-0 (PMC10283299; doi:10.1186/s12909-023-04384-0)
Supplement: Supplementary file 1 — Additional file 1. Questionnaire. Survey of medical students’ attitudes toward overseas experience. [file 12909_2023_4384_MOESM1_ESM.docx]

**Questionnaire:** Survey of medical students’ attitudes toward overseas experience

Based on the results of this survey, we plan to conduct as many workshops as possible to address your needs and share useful information. Thank you in advance for your consideration.

1. E-mail address
2. Name of your institution
3. School Year
4. Which of the following specialties are you interested in^*1^?

Multiple answers

□ Internal medicine

□ Surgery

□ Pediatric medicine

□ Family medicine

□ General medicine

□ Emergency medicine

□ Infectious disease practice.

□ Anesthesiology practice

□ Public health (MPH)

□ Radiology practice

□ Cardiac surgery practice

□ Others

1. Are you interested in working as a physician abroad?

1: Not at all~ 5: Very high

1. How committed are you about working as a doctor abroad?

1: Not at all~ 5: Very high

1. How are you preparing to work abroad?

Multiple answers

□ Considering studying or training abroad

□ General English language study

□ Studying medical English, for example, by preparing for USMLE.

□ Participating in lectures delivered by doctors with experience abroad

□ Collecting information from the Internet and other resources.

□ Nothing

□ Others

1. Please provide the name of any student community or study groups you have previously belonged to in order to prepare for the type of preparation you have indicated above.
2. Which type of international career are you interested in?

Multiple answers

□ International organization

□ International NGOs

□ Clinical training abroad (short-term, during medical school)

□ Clinical training abroad (residency or fellowship)

□ Research abroad

□ Health officer at a Japanese governmental organization (e.g., Japanese embassies in foreign countries)

□ Professional at the Japan International Cooperation Agency.

□ Only thinking about working in Japan

□ Others

1. Which region are you interested in working in?

Multiple answers

□ North America

□ South America

□ Europe

□ Africa

□ Asia

□ Oceania

□ Japan

□ Others

1. What is your reason for hesitating to work abroad?

Multiple answers

□ Do not know the career options.

□ Lack of role models

□ Lack of peers who share the same goal

□ Language barriers

□ Cannot work in domestic or international settings.

□ Obtaining foreign medical licenses

□ Others

1. What kind of projects are you interested in?

Multiple answers

□ Lectures by doctors working abroad with question-and-answer sessions.

□ Study groups to understand the experiences of doctors of clinical study abroad.

□ Medical English study groups

1. The Student Section of the Japanese Chapter of the American Board of Internal Medicine aims to plan study groups and lectures based on the results of this survey. Would you like to receive email notifications of future events?

Yes/No

Note 1: The fields were categorized based on the "Series on US-Japan Medical Exchange" edited by the Japan-North America Medical Exchange Foundation.
